# Supplementary material for: Multiscale segmentation using hierarchical phase-contrast tomography and deep learning
Source: PLoS Comput Biol. 2026 Feb 2;22(2):e1013923. doi: 10.1371/journal.pcbi.1013923 (PMC12880754; doi:10.1371/journal.pcbi.1013923)
Supplement: S1 Text — (PDF) [file pcbi.1013923.s001.pdf]

Supporting Information for: Multiscale Segmentation using Phase-contrast Tomography and Deep Learning

Yang Zhou[1], Shahab Aslani[2,3], Yousef Javanmardi[1], Joseph Brunet[1,9], David Stansby[1,4], Saskia Carroll[1], Alexandre Bellier[5], Maximilian Ackermann[6,7,8], Paul Tafforeau[9], Peter D. Lee[1], Claire L. Walsh[1*]

1 Multiscale X-ray Imaging (MXI) Lab, Department of Mechanical Engineering, University College London, London, UK
2 Satsuma Lab, Hawkes Institute, University College London, London, UK
3 Department of Respiratory Medicine, University College London, London, UK
4 Advanced Research Computing Centre, University College London, London, UK
5 Univ. Grenoble Alpes, Department of Anatomy (LADAF), AGEIS, CIC INSERM 1406, Grenoble, France
6 Institute of Anatomy, University Medical Center of the Johannes Gutenberg University Mainz, Mainz, Germany
7 Institute of Pathology, Uniklinik RWTH Aachen, Aachen, Germany
8 Institute of Pathology and Department of Molecular Pathology, Helios University Clinic Wuppertal, Wuppertal, Germany
9 European Synchrotron Radiation Facility, Grenoble, France

∗ c.walsh.11@ucl.ac.uk

Contents

1 HiP-CT tomographic information **2**

2 Data pre-processing **3**

3 HiP-CT multiscale registration **4**

4 Training on complete organ scans **5**

5 Prediction post-processing **7**

6 HiP-CT training data **11**

7 Results of LADAF-2021-17 right kidney **12**

1 HiP-CT tomographic information

Table A summarises the key scanning parameters for each sample and voxel size used in this study. The reconstructed datasets are available for download via the DOI links (cited as references) provided in the Dataset section of the main manuscript. Additionally, the portal **human-organ-atlas.esrf.eu** offers in-browser visualisation using Neuroglancer and access to supplementary data, including metadata, donor medical information, and image reconstruction parameters.

Table A. HiP-CT scan parameters

Organs	Voxel size (μm)	Data label	Acquisition mode	Projection number	Projection distance (m)	Attenuators	Average energy (keV)	Surface dose rate (Gy/s)	Number of scans	Per scan time (min)
S-20-28	2.5	VOI-01,<br>VOI-02,<br>VOI-03,<br>VOI-04,<br>VOI-05,<br>VOI-06,<br>VOI-07,	Half	6000	1.44	Al 0.51mm,<br>Mo 0.24mm,<br>SiO2 16mm<br>rods 4x4mm	~81	U.N	23,<br>17,<br>8,<br>5,<br>5,<br>9,<br>5	U.N
LADAF-2020-17<br>Left Kidney	2.6	VOI-01.1,<br>VOI-02.1	Half	6000	1.44	Mo 0.23mm,<br>SiO2 40mm<br>rods 10x4mm	~83	35	15,<br>17	5.3
LADAF-2020-17<br>Right Kidney	2.6	VOI-01.1,<br>VOI-02.1,<br>VOI-03.1	Half	6000	1.44	Mo 0.23mm,<br>SiO2 40mm<br>rods 10x4mm	~83	35	49,<br>8,<br>24	5.3
	6.5	VOI-01,<br>VOI-02,<br>VOI-03,	Half	6000	3.5	Mo 0.23mm,<br>SiO2 40mm<br>rods 10x4mm	~83	35	44,<br>13,<br>35	U.N
	25.0	Complete organ	Half	6000	3.5	Mo 0.1mm,<br>SiO2 32mm<br>rods 8x4mm	~81	U.N	U.N	4.3
LADAF-2020-27<br>Left Kidney	1.29	Central column	Half	6000	0.5	Al 2mm,<br>Mo 0.1mm,<br>SiO2 bars<br>3*5mm	~74	161	6	12
	6.05	Central column	Half	6000	3.475	Al 2mm,<br>Mo 0.1mm,<br>SiO2 60mm<br>rods 12x5mm	~89	10.5	13	5
	25.08	Complete organ	Half	6000	3.475	Al 2mm,<br>Mo 0.1mm,<br>SiO2 60mm<br>rods 12x5mm	~93	10.5	50	2.5

2 Data pre-processing

Data pre-processing is a crucial step for preparing HiP-CT datasets before training neural networks. As illustrated in Fig A (A), the raw HiP-CT data are 16-bit with a narrow and sample-dependent intensity range, which results in insufficient contrast between the organs and backgrounds. Additionally, training with 3D 16-bit volumes, even after cropping into smaller 3D patches, presents computational challenges. To address these issues, we applied 3D Contrast-Limited Adaptive Histogram Equalisation (CLAHE) to the 16-bit data volume first to enhance contrast and normalise intensity distribution. Then, the processed data were converted to 8-bit format, reducing memory requirements and improving training efficiency. The pre-processed sample image is shown in Fig A (B).

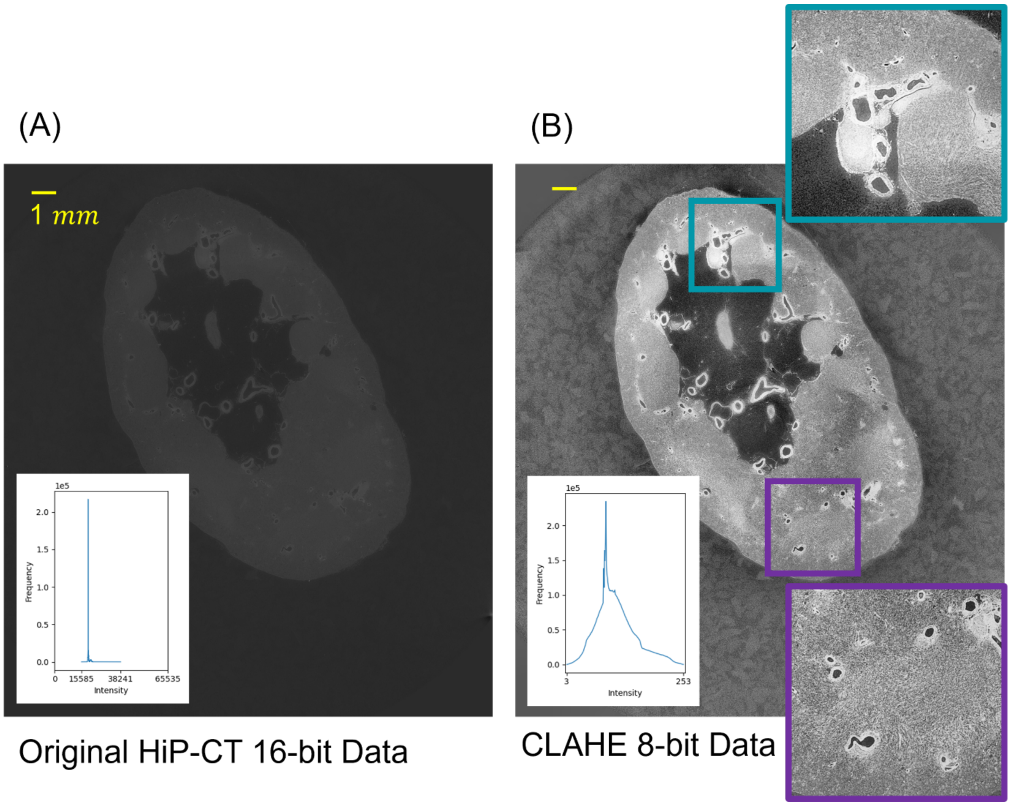

Fig A. HiP-CT (A) original 16-bit sample slice, compared to (B) pre-processed image using CLAHE and 8-bit conversion. The zoomed regions (blue and purple squares) show the highlighted textures and features after applying CLAHE.

3 HiP-CT multiscale registration

Multiscale registration is a key component of the proposed segmentation pipeline, enabling the generation of pseudo-labels and training datasets at lower resolutions. As shown in Fig B (A), a representative 2D slice from the complete kidney scan of LADAF-2020-27 at $25.08\mu m$/voxel resolution is displayed. Fig B (B) illustrates the result of aligning this low-resolution slice as a fixed image with the corresponding higher-resolution counterpart as a moving image at 12.1 μm/voxel, demonstrating the effectiveness of the registration across scales.

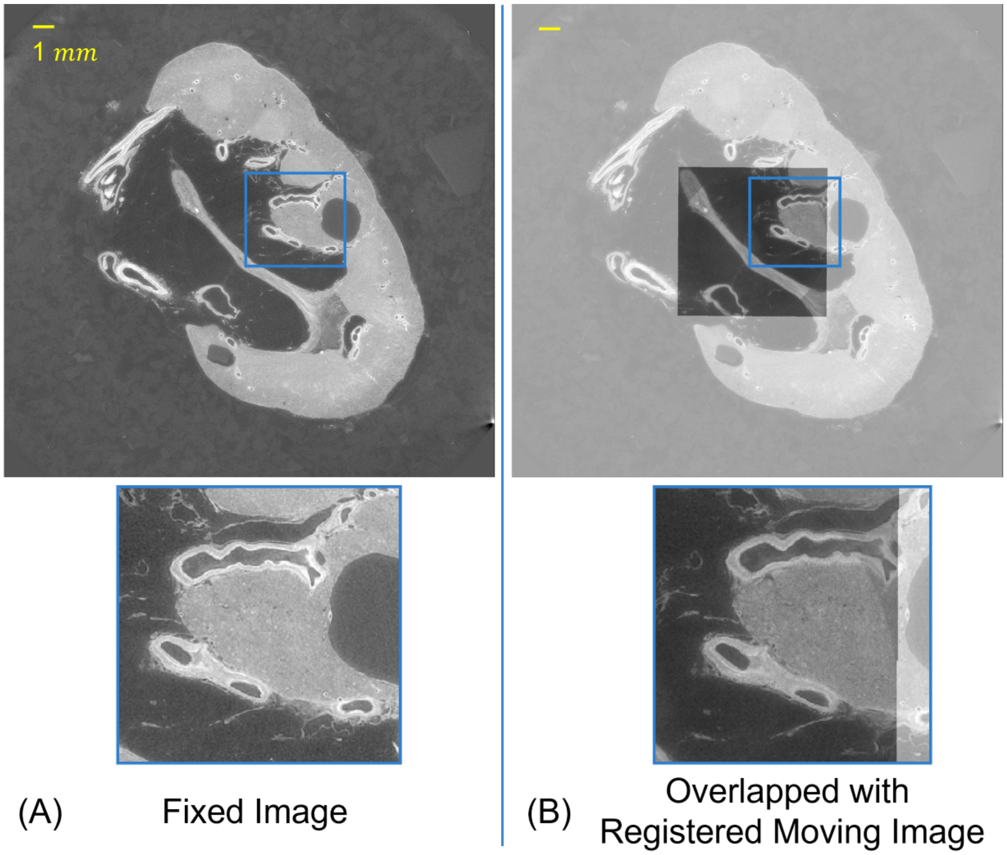

Fig B. Registration between two resolutions of HiP-CT data. (A) Sample was from LADAF-2020-27 left kidney, complete organs at 25.08 μm/voxel. (B) Registered sample slice from intermediate-resolution column at 12.1 μm/voxel (binned by 2 from the original column at 6.05 μm/voxel), overlay on the corresponding 25.08 μm/voxel slice. The overlay region is shown in dark, and the padding region is in white. The blue squares on (A) show the original region at 25.08 μm/voxel, compared to the one on (B) with an overlay registered slice at 12.1 μm/voxel.

4 Training on complete organ scans

As discussed in the main manuscript, training on complete organ scans at low resolution (25.08 μm/voxel) is challenging due to the image degradation. Fig C presents the original low-resolution HiP-CT 2D slice alongside the corresponding CLAHE-enhanced slice and annotation from the training data of LADAF-2020-27 left kidney. Although CLAHE improves overall contrast and enhances visibility of structural boundaries, glomerular features remain substantially degraded at this resolution, making it challenging for the model to distinguish them reliably. Therefore, to improve the segmentation performance and the Dice scores, we investigated several training strategies as shown in Fig D.

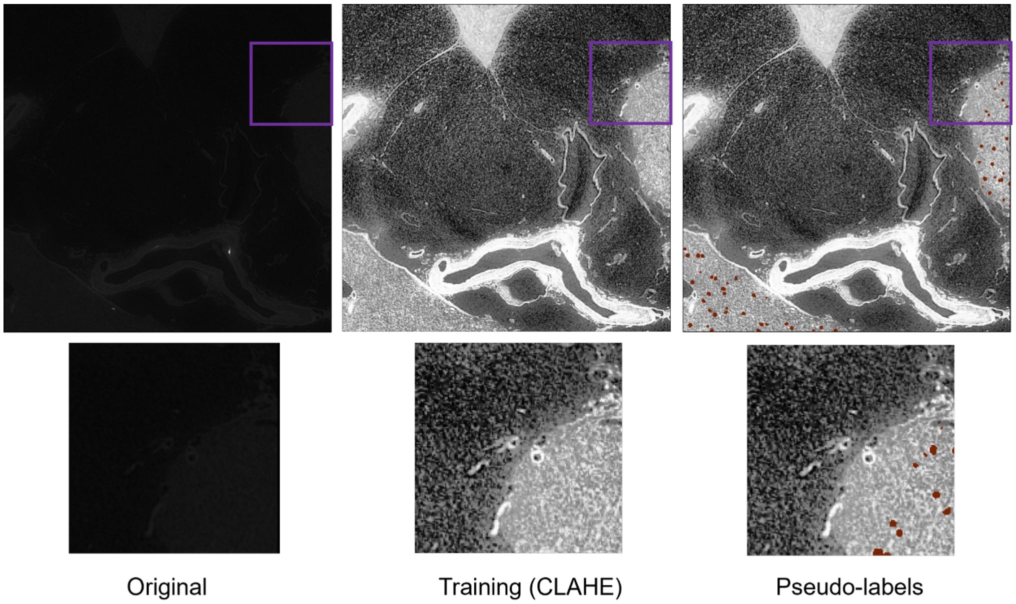

Fig C. The 2D slices from complete organ scan of LADAF-2020-27 left kidney at 25.08 μm/voxel. The original image, the training image after CLAHE applied and the pseudo-labels from the predictions of the previous resolution in the multi-scale segmentation pipeline are shown.

We first compared fine-tuning versus training from scratch, as in panel (A) of Fig D. The results show that fine-tuning yields a higher Dice score after training 1000 epochs, indicating better performance. Given the low-resolution nature of the data, where pseudo-labelled glomeruli normally occupy a small fraction of the volume, we also sought to reduce background dominance. To do so, we excluded 3D patches in which glomerular labels comprised less than 1% of the volume. Panel (B) shows a comparison between training on the full dataset versus the filtered datasets without low-label patches over 1000 epochs. We found that removing these nearly empty cubes significantly improved model performance. Given the similar performances between the trainings on the datasets of removing all the cubes with labels volume smaller than 1%, and keeping 0.7% of those cubes, we used the latter for training on the low-resolution whole kidney data.

Despite these improvements, Dice scores seemed to continue to rise beyond 1000 training epochs, indicating under-convergence. Therefore, we explored extended training epochs with different learning rate scheduling, as shown in panel (C). Two learning rate

decay strategies were tested: polynomial decay (Eq. 1) and exponential decay (Eq. 2):

$$LR_{poly} = \begin{cases} 0.01 \times (1 - \frac{x}{1000})^{0.9}, & \text{if } 0 \leq x < 1000, \\ 0.002 \times (1 - \frac{x}{1500})^{0.9}, & \text{if } 1000 \leq x < 1500, \end{cases} \tag{1}$$

$$LR_{exp} = 0.01 \times 0.994^{x}, \tag{2}$$

where LR_{poly} and LR_{exp} denotes the learning rate at epoch x for polynomial and exponential decay, respectively. Considering that the Dice score increased very slowly around 1000 epochs in previous experiments, both polynomial and exponential learning rate schedules were designed to be small when continuing training from 1000 epochs to avoid the overfitting problem. While exponential decay accelerated convergence, polynomial decay consistently led to better segmentation performance. Additionally, extending training to 1500 epochs provided only a slight improvement, suggesting that further training beyond this point would likely involve a tradeoff between performance gain and computation cost. Therefore, the final model for low-resolution data was trained using polynomial learning rate decay over 1500 epochs.

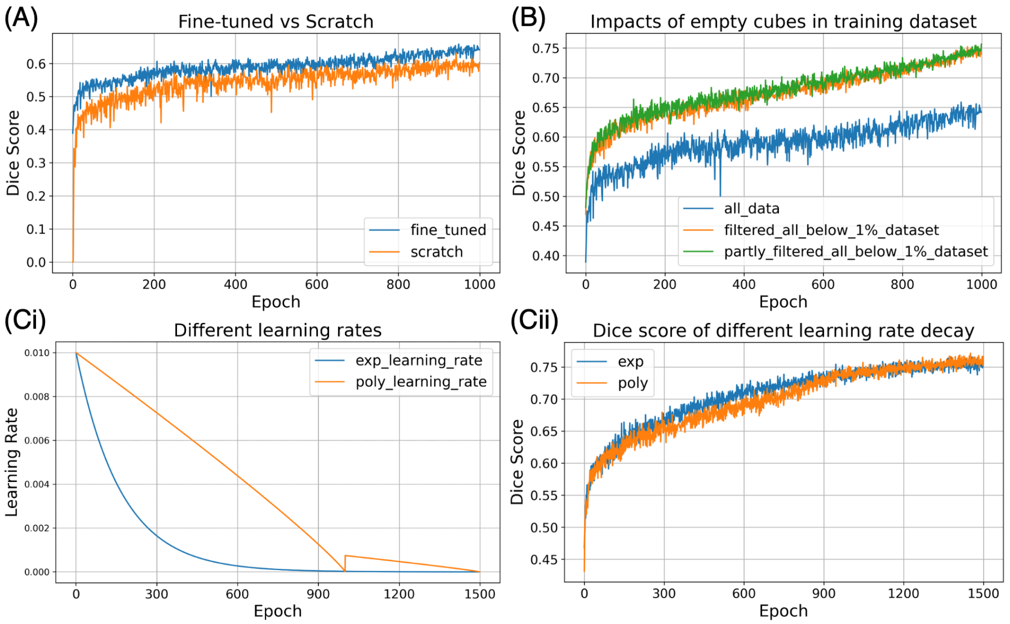

Fig D. Evaluation of different training strategies for low-resolution complete organ scans at 25.08 μm/voxel.(A) Comparison between a fine-tuned model initialised from intermediate-resolution training and a model trained from scratch using nnUNet defaults. (B) Performance comparison between training on all registered cubes (blue line), on a subset of all the cubes with label volume smaller than 1% filtered (orange line), and a subset keeping 0.7% of the cubes with label volume smaller than 1% (green line). (Ci) Polynomial and exponential learning rate decays over 1500 epochs investigated in this study. (Cii) Training Dice scores of polynomial and exponential learning rate decays among 1500 training epochs.

5 Prediction post-processing

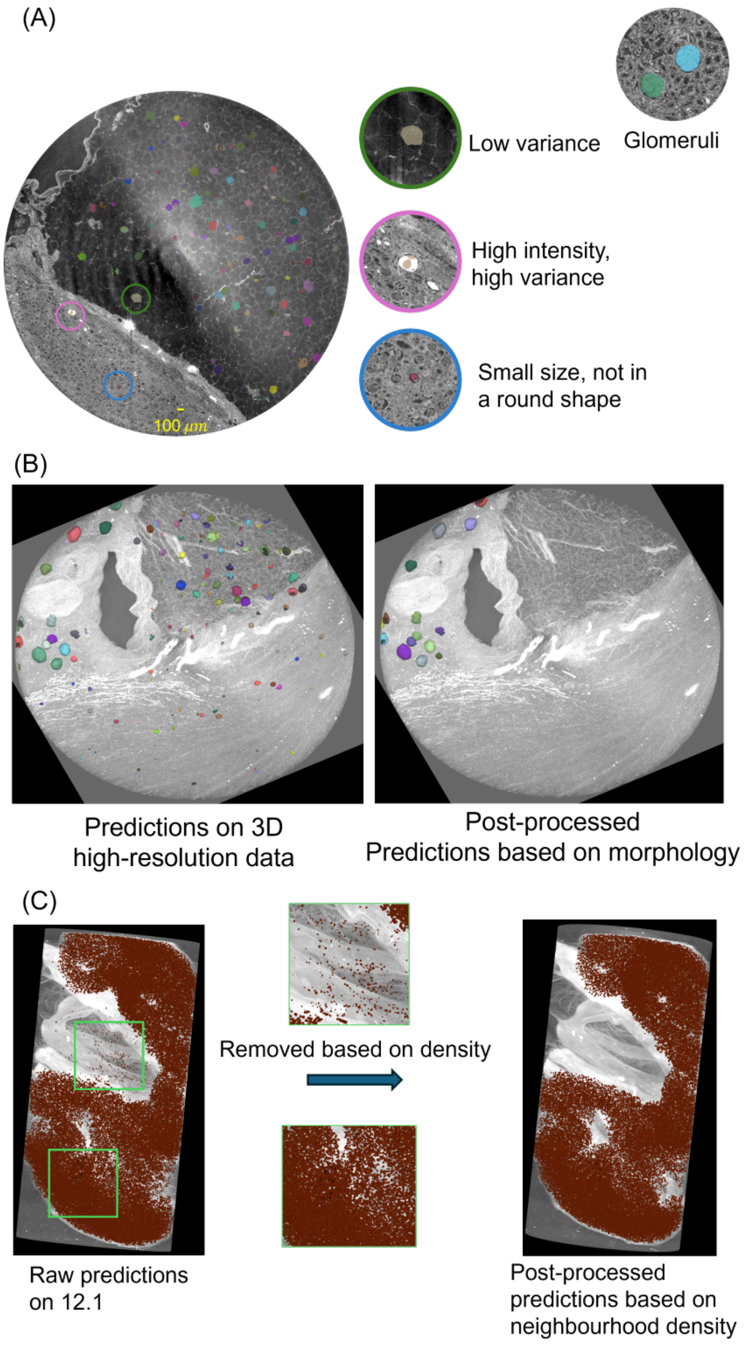

Fig E. Effects of post-processing on segmentation predictions. (A) Examples of false positives in high-resolution data (LADAF-2020-27 Left Kidney, 2.58 μm/voxel) that can be eliminated using intensity-based and morphological criteria. (B) Segmentation result after applying post-processing to high-resolution data. (C) False positives in intermediate-resolution data (12.1 μm/voxel) that require an additional density-based filtering step for effective removal.

The effect of the post-processing technique is shown in Fig E. Panels (A) and (B) show results after post-processing on high-resolution data at 2.58 μm/voxel. At this resolution, false positives, classified into three categories as described in the main manuscript, can be effectively removed using the thresholding parameters based on intensity and morphological properties such as intensity variances and glomeruli size. However, for lower-resolution data, as shown in Fig E (C), these parameters alone are insufficient. Therefore, an additional post-processing step based on prediction density is introduced to further reduce false positives. Fig F further illustrates the glomerular predictions and false positives in the whole intermediate VOIs. Compared to Fig E (A), the false positives in the intermediate resolution have lower voxel intensities and are spatially coarser. Therefore, to apply the post-processing, we used different hyperparameters.

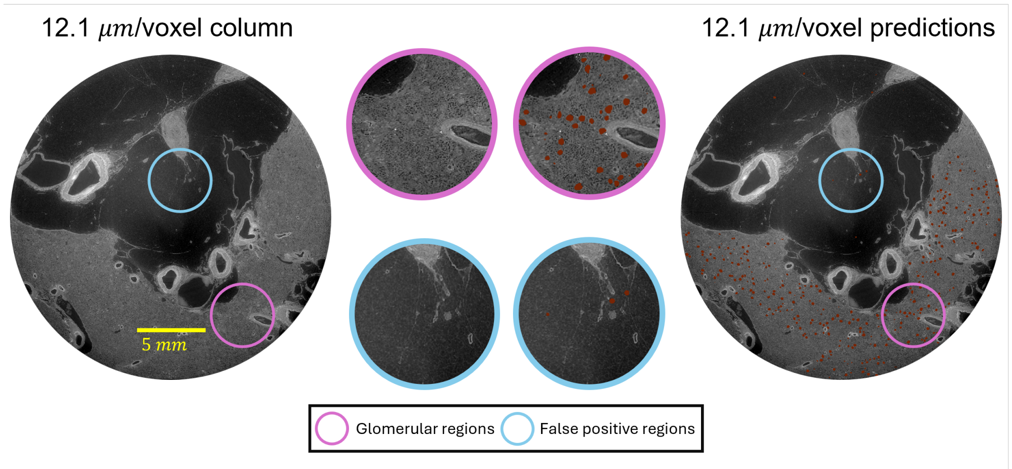

Fig F. Detailed false positives at intermediate-resolution (12.1 μm) complete VOIs from LADAF-2020-27 left kidney. It presents two regions from intermediate-resolution VOI, showing the glomerular regions (light blue) and false positive regions (light purple).

To determine optimal post-processing parameters, we computed the morphological tables, including intensities, sizes, shapes, locations etc., and then 20 sets of thresholds were generated for each resolution using Latin Hypercube Sampling (LHS). Fig G presents the results of hyperparameter search among high-resolution and intermediate-resolution VOIs of LADAF-2020-27 left kidney. The figure shows the average Dice before applying post-processing (dashed lines), and the average Dice after applying the hyperparameters in each search (bar charts), evaluated on all training cubes, including one additional empty cube per resolution. Among the generated threshold sets with the same performance, the selection criterion is to preserve as many true positives as possible while eliminating false positives.

For high-resolution data at 2.58 μm/voxel, the false positives include fat area (low variance) and blood clot areas (high variance), as shown in Fig E (A). Therefore, we applied the LHS to search the variance range for true positives, formed by a lower bound and an upper bound. The LHS sampling was conducted within the percentile of a sorted intensity list, where the variance lower bound was sampled from 0% to 30%, while the variance upper bound was sampled from 70% to 100%. The roundness used for high-resolution false positive removal was sampled from a range of 1% to 99%. The blue panel in Fig G shows the results where search 5 and search 12 achieved the best average Dice of 0.934, exceeding 0.734 before post-processing was applied. To preserve more true positives, a larger variance range from search 12, as shown in the scattering plot, was selected.

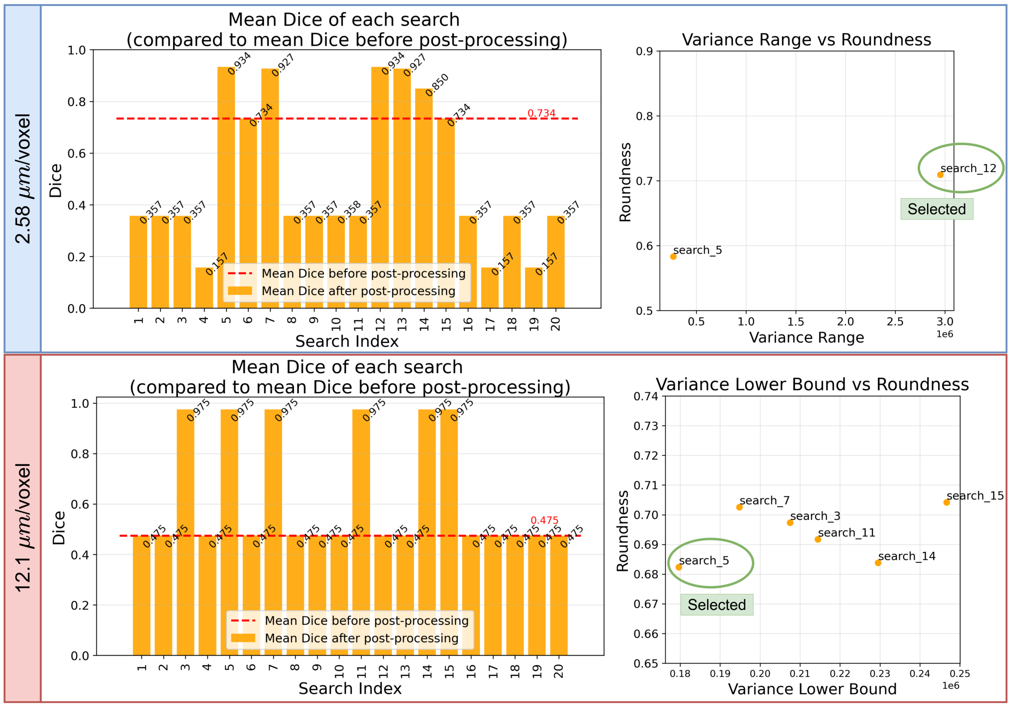

Fig G. Selection of post-processing hyperparameters using Latin Hypercube Sampling (LHS). The bar chart displays the mean Dice for each hyperparameter set sampled via. LHS, validated by all the training cubes (involving an additional empty cube) as validation cubes. The horizontal line indicates the mean Dice (in red) across all the validation cubes before applying any post-processing. The scatter plots illustrate how the optimal parameter set was selected among those with the same performance, prioritising thresholds that preserve more true positives. For example, in 2.58 μm data, the search with a variance range (search_12) preserves a larger amount of true positives and was selected. As for 12.1 μm data, the search with a lower variance lower bound (search_5) can keep as much as true positives which was selected.

In contrast, for the intermediate-resolution data at 12.1 μm/voxel, the false positives in the fat area present with lower intensity compared with the high-resolution 2.58 μm/voxel VOIs due to the resolution constraints and different scanning parameters, resulting in brighter cortical tissues. Therefore, we only sampled the lower bound of intensity variance, instead of calculating the range. Due to the false positives being darker than the cortical area, and the glomeruli being small, which only take a few voxels in the 12.1 μm, we sampled the lower bound within the percentile of 0% to 3%, roundness within 0.1% to 0.3%. The results are shown in the red panel in Fig E. The search results present only two categories, one with Dice 0.475, the same as the Dice before applying post-processing and another with Dice 0.975. This was caused by smaller search ranges for variance and roundness. However, these smaller ranges improve the search efficiency to obtain enough optimised hyperparameter pairs for selection. On the one hand, enlarging the search range for the variance lower bound results in a higher value sampled to over-perform on the cortical area, due to there is a clear intensity gap from dark fat area to bright cortical area in this resolution. On the other hand, with the imaging resolution decrease and glomeruli taking fewer voxels, the predicted roundness tends to be in a small range. Using a larger roundness search range, post-processing can also over-perform. These can be resolved by setting more search trials, but this is inefficient. Therefore, manually setting a lower search range can help

obtain enough optimised hyperparameter pairs, as shown in the red panel in Fig G. From the 6 optimised hyperparameter pairs, the search 5 with a lower variance lower bound preserved most true positives was selected. After that, as illustrated in the main manuscript Method section, a density-based 3D filter was applied as a complement alongside the smaller search range to remove the coarse false positives that share similar variance as true positives in the intermediate resolution data, such as blood clot area.

6 HiP-CT training data

Fig H illustrates the extracted 2D slices from the training data of LADAF-2020-27 left kidney. The 2D visualisations here are displayed at spatial dimensions of 512×512 for high resolution, 284×284 for intermediate resolution, and 657×657 for low resolution, respectively, preprocessed by 3D CLAHE before cropping as training patches.

During scanning, high-resolution volumes were normally selected from cortical regions, which are densely populated with glomeruli. These regions are within the intermediate-resolution column. To ensure effective threshold selection during the post-processing stage, particularly for false positive removal, it was necessary to introduce empty cubes (i.e., volumes without glomeruli) at both high and intermediate resolutions for effective validation.

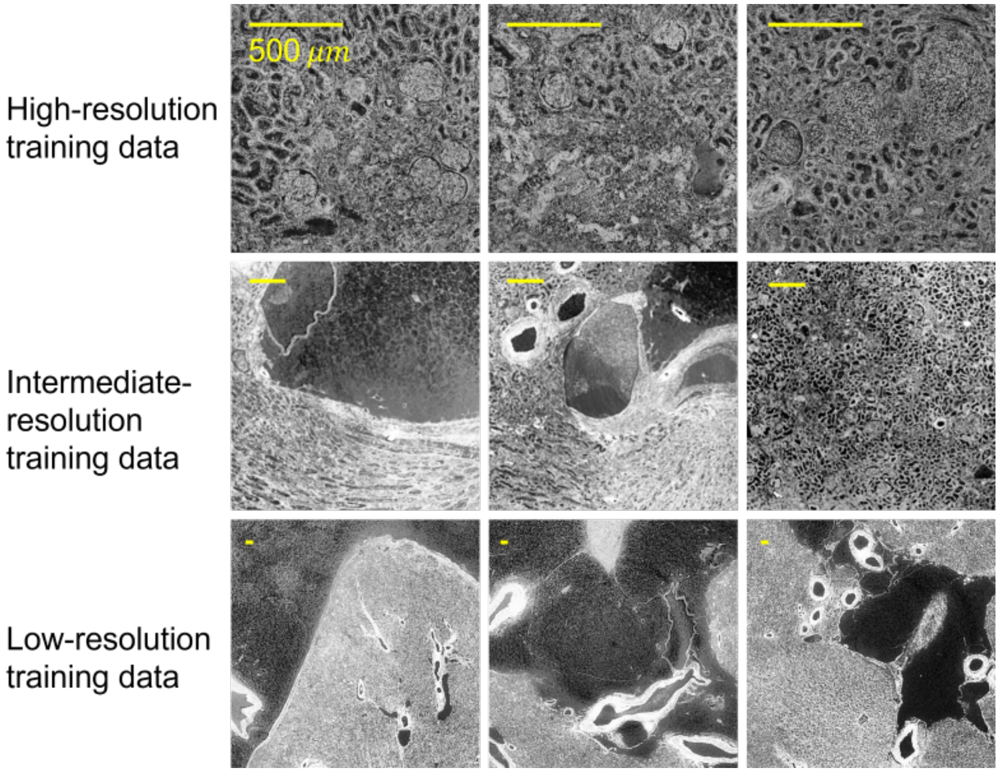

Fig H. Training data visualised in 2D extracted from LADAF-2020-27 left kidney data imaged by HiP-CT. The high-resolution data (2.58 μm/voxel) and intermediate-resolution data (12.1 μm/voxel) do not involve much fat area, so they require additional empty cubes to evaluate the post-processing effects. However, the low-resolution data (25.08 μm/voxel) has a larger field of view.

7 Results of LADAF-2021-17 right kidney

This section presents the multiscale segmentation results for the LADAF-2021-17 right kidney, which was used for morphological analysis. For this kidney, the intermediate-resolution volumes were at 13 μm/voxel, and the low-resolution complete kidney was acquired at 25 μm/voxel. We applied the high-resolution model trained on manually annotated data to the VOI-02 and VOI-03. These volumes were subsequently registered to the low-resolution complete kidney volume to generate the training data for fine-tuning the model.

As described in Fig 3A from the main manuscript, 5-fold cross-validation of nnUNet on the LADAF-2020-27 left kidney did not show significant variation across folds. Therefore, for LADADF-2021-17 right kidney, we only implemented fold 0 training and used the model for glomeruli segmentation to streamline the workflow. Table B shows the number of training and validation patches used for fine-tuning at each resolution, with a 9:1 train/validation split. This table also reports the best Dice score achieved during the fine-tuning processes. Fig I further shows the detailed losses and validation Dice scores among training on 13 μm data and 25 μm data, respectively.

Table B. LADAF-2021-17 Right Kidney training dataset sizes and best Dice scores during training for each resolution.

	Dataset size (patches)		Best Dice scores	
	Training	Validation	Training	Validation
13 μm/voxel	1498	375	0.935	0.915
25 μm/voxel	1556	390	0.803	0.805

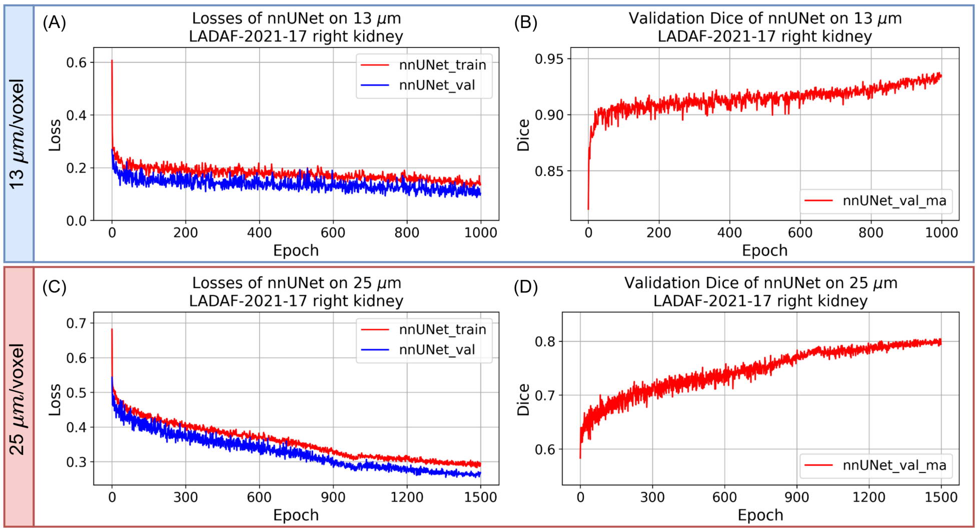

Fig I. Training losses and validation Dice scores after exponential moving average at each resolution fine-tuning for the LADAF-2021-17 right kidney sample. (A) The losses of training and validation datasets during training on 13 μm data. (B) The validation Dice during training on 13 μm data. (C)The losses of training and validation datasets during training on 25 μm data. (D) The validation Dice during training on 25 μm data.
