# Supplementary material for: Multiscale segmentation using hierarchical phase-contrast tomography and deep learning
Source: PLoS Comput Biol. 2026 Feb 2;22(2):e1013923. doi: 10.1371/journal.pcbi.1013923 (PMC12880754; doi:10.1371/journal.pcbi.1013923)
Supplement: Attachment — Submitted filename: Response_to_reviwers.pdf [file pcbi.1013923.s003.pdf]

Re: Decision from PLOS Computational Biology submission PCOMPBIOL-D-25-01047

Dear Reviewers and Editor,

We are writing in response to the reviewers' comments on PLOS Computational Biology submission PCOMPBIOL-D-25-01047.

We would like to thank the reviewers and editor for their critical feedback and constructive suggestions. We have thoroughly addressed all the comments in the revised manuscript. Changes to the manuscript are highlighted in red text. Please see the attached revised version. Below, we provide detailed responses in blue corresponding to the comments in *italic*.

Reviewer #1

The authors present a computational study about kidney segmentation. Especially, they focus on building a tool that can be applied across a multitude of resolutions, because earlier tools can just be applied on the resolution they were trained on. The overall manuscript is well written and seems to be adding something new to the field. I think the manuscript is suitable for publication in PLOS Comp. Bio., but there are concerns from my side that need to be resolved beforehand. These are about:

- *The Dice_I that is introduced in the manuscript*
- *The analysis of which models are overfitting*
- *The definition/thresholding of the three zones in downstream analysis*
- *The availability of the code*

Thank you for the constructive comments, and please see the following responses.

Major

P. 9 methods/Post-processing/Dice_I

1. I *really do not get the point of the Dice_I score. I mean it is just binarizing the Dice score in three ordinal categories. Your argument is that "precise predictions of the blurry boundaries of glomeruli in low-resolution data are difficult". But then the solution should not be to make the evaluation metric "easier" by binarizing, right? I think some discussion about the Dice_I should be included in the discussion or methods section to explain a little bit more why the Dice_I is useful.*

   By comparing the Dice scores and the Instance Dice scores in post-processing, we agreed that the Dice scores can reflect the effectiveness of our post-processing method, and the Instance Dice scores provide similar information, which can be

redundant. Therefore, we have removed the Instance Dice scores from the manuscript.

2. *Also the Dice_I variable is introduced, but never used. It seems like "Instance Dice scores" in table 2 is Dice_I. If you introduce the Dice_I you should also use it in your manuscript. E.g. why is it not reported in Table3 anymore?*

   We have removed the Instance Dice scores. Please see the detailed reason in the first comment.

3. *When looking at table 2 the difference between Dice and Dice_I is ~+0.02 for 2.58 and 12.1 before and after correction. For 25.08 there is no notable difference. So is it not enough to just report the Dice? What additional information is given by reporting the Dice_I?*

   We have removed the Instance dice scores. Please see the detailed reason in the first comment.

<u>P. 9 methods/Post-processing/Dice_I: "[...] we propose the DiceI to indicate a higher overlap rate between predictions and ground truths, which helps perform density and distribution studies of the glomeruli on the low-resolution data."</u>

4. *What do you mean with "indicate a higher overlap rate"? I understand it as we correct our Dice score up to have numerically higher results. Wouldn't it be better to just use the regular Dice score and then explain that worse results are seen because of low-resolution data?*

   We removed the Instance Dice scores from the manuscript.

   Initially, we proposed the Instance Dice scores as we hypothesised that the predictions might not fully overlap with pseudo-labels in the lower-resolution data, where the glomeruli boundaries are not clear. Therefore, we set thresholds on the Dice score and push any Dice score greater than 0.8 to 1. However, as the reviewer pointed out, the proposed Instance Dice scores and Dice scores reported in the manuscript were very close. Therefore, using Dice scores solely is sufficient to evaluate the post-processing effectiveness.

   As recommended by the reviewer, we added a section to the Results/Post-processing effects (page 12, line 341-line 347) to provide more details on the reduced performance on low-resolution data.

P. 11 results/Glomeruli segmentation on high-resolution labelled data: “However, for UNETR and SwinUNETR, the training losses decreased while the validation losses increased slightly at the end. Similar patterns were observed in the rise of the Dice scores. These indicated that the UNETR and SwinUNETR experienced the over-fitting problem, achieving better training performance on the training dataset but performing worse on the unseen data.”

5. *I agree that UNETR is overfitting, but SwinUNETR is as much overfitting as nnUNet is. What I mean is that for SwinUNETR AND nnUNet train is larger than validation. I would argue that only UNETR is really overfitting. SwinUNETR is in the same range as nnUNet and still ok in the sense of overfitting*

   Thank you for the constructive comment. We attached a zoom-in version of the figure for Panel (B) below. The nnUNet validation dice (dotted yellow line) closely tracks the training loss (solid yellow line). In contrast, SwinUNETR’s validation loss (dotted blue line) remains around 0.2 as the training loss (solid blue line) decreases.

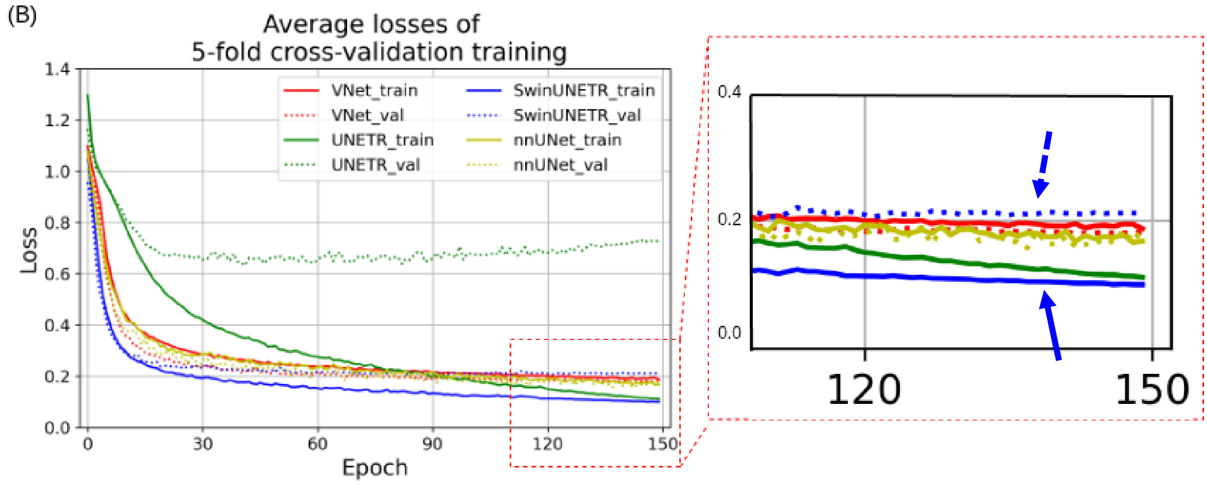

   In this case, we agree with no obvious overfitting for SwinUNETR, as there isn’t a ‘breaking point’[1] where the validation loss starts to increase. We have updated the text and noted that only UNETR is overfitting on page 11, line 297 to line 309.

   [1] Aliferis, Constantin, and Gyorgy Simon. "Overfitting, underfitting and general model overconfidence and under-performance pitfalls and best practices in machine learning and AI." Artificial intelligence and machine learning in health care and medical sciences: Best practices and pitfalls (2024): 477-524.

6. *You could make this paragraph more precise by actually using numerical differences instead of “decreases”, “increased slightly”, “in the rise of”, “but performing worse on the”. This is what I mean: As training progressed, both the training and validation*

losses for nnUNet and VNet decreased [BY XXXX%]. However, for UNETR and SwinUNETR, the training losses decreased [BY XXX%] while the validation losses increased slightly [BY XXXX] at the end [AFTER EPOCH XXX]. Similar patterns were observed in the rise [BY XXXXXX] of the Dice scores.

We have modified the paragraph on page 11, line 297 to line 309. We provided more statistics and comparisons.

P. 15 results/Morphological analysis: "Despite the overall uniformity in volume distribution across the cortex, segmenting the cortex into three zones—inner (0–0.33), mid (0.33–0.66), and outer (0.66–1.0)—revealed subtle regional differences."

7. *These three zones are defined by percentiles and right now I think they lack some more elaborate explanation. Why do you select 3 zones and not e.g. 2 or 4? Either define clearly that they were defined by the 33- and 66-percentile. Alternative and probably more robust way would be to define the number of zones and then the thresholds of the zones by calibration. Also it would be necessary to perform a statistical test to check for the significance of the mean differences. Maybe these zones also need to be defined per patient? For the male kidney I see a peak of glomeruli size between 0.0-0.1 and for the female it is more at ~0.2*

Three zones are universally used in current anatomical renal studies, like in [1,2], which correspond to juxtamedullary, mid-cortical, and superficial regions. To align with the studies, we re-divided three zones based on glomerular diameters in each kidney and restructured the morphological studies. The update section on page 14, line 398 to page 16, line 464. The summary of key changes is as follows:

- Three zones were divided by 3 times of mean glomerular diameters from each kidney (205.14 μm for LADAF-2020-27 left kidney and 182.68 μm for LADAF-2021-17 right kidney). The division follows the study of [1]. This tripartite division strategy was defined according to glomerular size from each kidney.
- Regenerate Figure 7 (page 16) with new "proportions of cortical zones" showing the regional size of each zone, and "glomerular volumetric distribution violin plot" showing the glomerular volume distributions across three zones.
- A further morphological study for intermediate-resolution VOIs was added. Higher segmentation accuracy was achieved in the intermediate-resolution data, so adding them further reliably supported the morphological analysis of the low-resolution complete kidneys.
- Regarding the statistical analysis, zonal volumetric differences were assessed using the Kruskal–Wallis test across the three cortical zones. Test results confirmed that highly significant zonal differences in glomerular size

(p<0.001) for both complete kidneys in low-resolution and intermediate-resolution VOIs

[1] Samuel, Terry, et al. "Determinants of glomerular volume in different cortical zones of the human kidney." Journal of the American Society of Nephrology 16.10 (2005).

[2] Denic A, et al. Tubular and glomerular size by cortex depth as predictor of progressive CKD after radical nephrectomy for tumor. Journal of the American Society of Nephrology. (2023).

8. *The mean glomeruli are in the text around 10**6 micrometer**3. In figure 7, y-axis label it says 10**7, please use the same scale and also add to the y-axis label the unit (what is size of glomeruli, probably micrometer**3).*

   We have regenerated Figure 7 on page 16 for the morphological study. In the latest figure, the scales of the y-axis labels are aligned. The unit for the glomerular volume was also added to the y-axis labels.

9. *In the discussion (page 18) you name the "juxtamedullary, mid-cortical, and superficial regions". Are those the three zones that you refere to? Maybe try to make this connection more clear. Are there in the literature thresholds for these three zones? Maybe you can use these instead of the 33- and 66-percentile.*

   In the latest morphological analysis section, on page 15, first paragraph starting from line 407, we align the inner, middle and outer zones in our manuscript to "juxtamedullary, mid-cortical, and superficial regions". The previous studies divided these three zones based on anatomical features on the histological slices without a standard distance threshold. Therefore, we re-divided these three zones as in study [1] based on three times of glomerular diameters.

   [1] Samuel, Terry, et al. "Determinants of glomerular volume in different cortical zones of the human kidney." Journal of the American Society of Nephrology 16.10 (2005): 3102-3109.

10. *Add the three zones to the plots (C.1) and (C.2) of figure 7*

    We have removed the original figures that were based on normalised distances. In the latest zonal partition figures, Figure 7, we presented the proportions of zonal regions, and each zone was clearly used as the x-axis in the figure panels.

<u>Github/Code:</u>

11. *The documentation of the code needs some work. You provide an empty section about "Folder structure" and no instructions about how to set up your repo. I would include a requirements.txt file or conda environment. I just saw that in the pyproject.toml there are the dependencies given. Then please provide some instructions on how to set them up.*

We have updated the pyproject.toml file and also updated the Python environment configuration section.

12. *It would also be cool to have a minimal example that is given in the repo to run your code. Maybe just 2 or 3 very small images from your experiments that allow someone to use your functions and make them explorable.*

Our model works on a 3D HiP-CT dataset, and very small cubes might not contain the glomeruli. Therefore, to provide the test case, we advise downloading any of the training data cubes in 512^3 dimension and perform model inference on them.

We have updated the inference guidance on GitHub 2025-zhou-hipct-hierarchical-segmentation/segmentation/S_README.md at main · UCL-MSM-Bio/2025-zhou-hipct-hierarchical-segmentation

13. *Where is some code to make the automatic annotations that you describe in figure 2? I basically only see code relevant for step (B) pre-processing in the repo.*

Thank you for your valuable comment. The pseudo-labelling code has been updated to the GitHub repository under *2025-zhou-hipct-hierachical-segmentation/ registration/correlation.*

14. *Also the post-processing seems to be a relevant step in your work. I do not find any code about the post-processing in the repo. Please add this.*

Thank you for your constructive feedback. We have updated the post-processing code on GitHub under 2025-zhou-hipct-hierachical-segmentation/ segmentation/post-processing.

Minor

P. 6 methods/Data aquisition

15. *Data aquisition should be "acquisition"*

We have corrected the misspelling on page 5, line 145.

16. *is the "S-20-28" image a private one? It does not have a citation in table1*

We are progressively updating and publishing the datasets. Now the S-20-28 is public, and the DOIs are updated in the manuscript on page 6.

17. *Most kidneys are from France and only one is from Germany. Were they imaged at the same site? So the German kidney was transported to the same facility in France, that's how I understand this section right now. Please specify.*

Yes, all the organs were transported and scanned at ESRF, although they were collected from different locations. We have added more information to the manuscript on page 6, line 155.

P. 7 methods/Figure 2

18. *"Mannul" please correct this spelling mistake in the figure and also check all other figures for similar spelling errors.*

We have looked up the whole manuscript and changed the misspelling 'Mannul' to 'Manual'.

P. 8 methods/data pre-processing: "Training the deep neural networks on such big datasets is challenging, and following preliminary experimentation, we found that conversion of the data from 16-bit to 8-bit after application"

19. *Are these preliminary experimentations seen in one of your other papers on this topic (e.g. Vasculature segmentation in 3D hierarchical phase-contrast tomography images of human Kidneys; Deep learning for 3D vascular segmentation in hierarchical phase contrast tomography: a case study on kidney.)? - If yes please cite.*

Yes, we have added the citation to the manuscript on page 6, line 182.

P. 9 methods/Deep neural network optimisation

20. *"a weighted Dice loss" what is the weight that is used? In equation 2 and the text I am told that you add the additional Dice loss with a weight beta. You also say that "beta is the weight of the Dice loss relative to the cross-entropy loss, and is set to 1 by default" But what is beta then set to during training? Is it the relative occurrence*

of the classes (glomeruli <-> not-glomeruli)? Is it a fixed value that you adapted during parameter optimization? If already written somewhere else in the manuscript and I could not find it please reference the corresponding section.

We set the $beta$ as 1 in all our experiments in the manuscript to balance the contribution from region overlap (dice) and pixel-level accuracy (cross-entropy). We modified the section on page 9, line 262 to 263.

P.10 results/Figure 3

21. *why do you not have the _train and _val for nnUNet? I think it is really difficult to compare the different models if not the same metric is applied? So either all with moving average on none with moving average. Also this fact should maybe be mentioned somewhere in the methods?*

    The nnUNet implementation [1] does not support the calculation of training dice over training. Instead, they calculate the pseudo dice on the validation dataset using an exponential moving average. Therefore, we reproduce the exponential moving average on other models.

    We have updated Figure 3, panel (C), on page 10 and added the moving average calculation in the same section, page 10, line 288 - 295

    To this comment, we also further revised Table 3 and Figure 6 to align with the moving average validation Dice scores.

    - In Table 3, the Dice scores on train and validation splits were calculated after the models of each fold were trained, so they were not the moving average scores. They are the average of each fold. The text is updated on page 13, lines 370-372.
    - In Table 3, we calculated and updated the average dice scores on the training data after the models were trained.
    - In Figure 6, we removed the panel (B): 12.1 µm validation Dice scores during 5-fold cross-validation training and (D): 25.08 µm validation Dice scores during 5-fold cross-validation training. These two figures duplicate the information in Table 3.

    [1] https://github.com/MIC-DKFZ/nnUNet

P. 12 results/Table2

22. *Isn't it weird that the postprocessing is helping so much for the 12.1micrometer case (+~100%), while for the lower and higher resolution the increase is not so much? Can you elaborate this somewhere? Maybe the masking used for 25.08 has an*

influence? Or it is something else, I am not an expert in kidney segmentation. Maybe the end of this paragraph would be good to add 1 or 2 sentences about this observation? After: "Dice score and instance Dice scores were highly improved for high and intermediate-resolution data, while slightly improved for the low-resolution complete organ data."

We have added a short paragraph on page 12, lines 342 – 348, to provide more details on why the post-processing works better on intermediate resolution data.

In the 12.1 μm data, the contrast of the 12.1 μm data allows relatively see the glomeruli from the surrounding tissues. Therefore, the false positives in the VOIs can be filtered out by the shape-based parameters. In addition, most of the false positives are in the fat area, which presents low intensity variance. Accordingly, intensity-based filtering is also effective. However, in 25.08 μm, the post-processing applies a kidney mask, and the neighbourhood density cannot fully remove the false positives in the cortical region due to the low contrast. Moreover, the model on 25.08 μm can only achieve a relatively 0.775 to 0.8 dice score on the training dataset over training. This indicates that some of the glomeruli cannot be predicted in 25μm, which cannot be predicted by the post-processing pipeline.

<u>P. 16 results/Figure 7: "(A.1) and (A.2) illustrate 3D rendering of predicted glomeruli and the density calculated by a mean kernel of the complete kidney (LADAF-2020-27-Left, Female, aged 94)"</u>

23. *Define better what is 1 and 2., E.g.: "(A.1) illustrates a 3D rendering of predicted glomeruli and (A.2) the density calculated by a mean kernel of the complete kidney (LADAF-2020-27-Left, Female, aged 94)" -> clearly differentiate between what is 1 and what is 2.*

    We have updated Figure 7 on page 16 by placing A.1, A.2, B.1, B.2 on the top left corner of the figure panels.

<u>*P.17 discussion: "To address this, we developed an automated post-processing technique at each scale to eliminate false positives [...]"*</u>

24. *Did you develop a post-processing pipeline for each scale or did you use different parameters depending on which scale is given. By reading the manuscript (especially Figure 5) I think you have the same pipeline, but with different parameters. But here it sounds like you have different pipelines. Please specify.*

Yes, the post-processing pipeline is fixed, but the parameters searched by Latin Hypercube Sampling depend on the scales. We have updated the sentence on page 18, line 513-514.

P.18 discussion: "reported that hypertension results in a 20–30% increase in glomerular volume, and our findings are consistent with this trend."

25. *Could you maybe define your difference between the glomerular volume also in percentage for the three zones between the male and female kidney? This makes it a lot easier to compare your results to the literature (Hughson et al.). Make it easy for the reader and do the math for them, e.g. add "(zone1: 25%, zone2: 35%, zone3: 45%)"*

Thank you for your helpful suggestion. We have added the zonal volumetric increase to the section on page 19, line 575 – 578.

Supplement/general

26. *The ordering of the sections in the supplement are not in the same order as they occur in the manuscript. Please make this in order. Right now it is 1, 3, 4, 6, 5, 2, 7.*

We have rearranged the sections in the Supporting Information according to their first occurrence in the main manuscript.

- Main manuscript page 6 line 158 -> Supporting Information section 1
- Main manuscript page 6 line 178 -> Supporting Information section 2
- Main manuscript page 9 line 239 -> Supporting Information section 3
- Main manuscript page 9 line 253 -> Supporting Information section 4
- Main manuscript page 12 line 324 -> Supporting Information section 5
- Main manuscript page 12 line 332 -> Supporting Information section 6
- Main manuscript page 14 line 385 -> Supporting Information section 7

Supplement/1 HiP-CT tomographic information: "The reconstructed datasets are available for download via the DOI links provided in the Dataset section of the main manuscript"

27. *there are no DOIs in the main manuscript in section Dataset. There are only references given in Table1*

The DOIs were cited as references in the Dataset section. We have indicated this in the Supporting Information section 1, on page 2, line 12.

Supplement/3 Data pre-processing (Fig. 2)

28. The labels (A) and (B) should be mentioned in the figure description or removed

The figure has been rearranged as Fig.1. We have updated the section and added descriptions for (A) and (B) in the image caption on page 3.

Supplement/4 Data pre-processing (Fig. 3)

29. The labels (A) and (B) should be mentioned in the figure description or removed

The figure has been rearranged as Fig.2. We have updated the figure caption to describe (A) and (B) on page 4.

Supplement 5 Prediction post-processing (Fig. 5)

30. Lower panel: red text on orange bars is not readable

The figure has been rearranged as Fig.7. We have updated the figure on page 9. The red text has been moved to the legend, which shows the Mean Dice before post-processing is applied.

31. Also the labels in red and blue text are too long

We have updated Fig. 7. The previous blue dot line indicates the Instance Dice before post-processing. Since we found that Instance Dice acted similarly to Dice score in our manuscript, we have removed the Instance Dice from the main manuscript, so the blue line has also been removed in the Supporting Information.

32. Lower right scatter plot: there seems to be two whitespaces before the "vs" in the title

We have updated the scatter plot in Fig. 7 with clearer titles.

33. X-axis scale of the two scatter plots is not the same, use either full written numbers or 1e6 annotation, but not both.

In Fig. 7, the x-axis of the two figures has been updated using the same scale.

34. Legend of barplots: write "Mean Dice_I Score" to be consistent with the Dice_I that you introduced

As above, in Fig. 7, we have removed the Instance Dice in the manuscript and use Dice as the evaluation metric only, so the Dice_I has been removed as well.

35. *The scores seem to be categorical (e.g. 12.1 micrometer has only two scores (0.5 and 1.0), is this expected? Looks a little bit artificial.*

Thank you for your insightful comment. The categorical results come from narrow search ranges we used for the variance lower bound search and the roundness search. The choices are based on the properties of HiP-CT intermediate-resolution images.

- Compared to 2.58 μm data, the 12.1 μm data present darker in the fat area and brighter in the kidney tissue, resulting in higher intensity variance gap between some of the false positives and true positives. This property causes the sampled parameter to either remove all the false positives in the dark fat area (with a higher Dice) or do nothing (with the same Dice as before post-processing). To justify this, we added a new figure, Fig.6 on page 8.
- As in Fig. 6, the predicted glomeruli on 12.1 μm VOIs are small, showing a narrow range of roundness.
- From the above discussion, although a larger search range is possible, it is not ideal and efficient to obtain enough hyperparameter pairs for comparison so that we can select the optimised pair. Otherwise, we will need to sample hundreds or thousands of rounds.
- In summary, due to the false positives being in darker areas and the predictions being small, we set a narrow search range so that we can efficiently sample enough hyperparameter pairs. However, it can result in categorical effects by either removing all false positives in the dark area or doing nothing. The density-based removal will act as a complement to further remove the coarse false positives, such as a blood clot, in the tissue area.

To justify these, we added a new figure (Fig.6) to show the false positives in 12.1 μm and a new section on pages 9 and 10

36. *Is the red line really the mean Dice score for 12.1 micrometer? It looks more like the median, because there are only bars on the same level as the mean line and some bars above. Therefore, the mean needs to be larger than 0.5 (maybe ~0.6).*

The red line was small in the previous figure, which confused readers. The red line represents the mean Dice **before** pre-processing, while each bar represents the mean Dice **after** post-processing by each parameter search. We plotted the red line to validate the effectiveness of the parameter search, making sure that the best

searches achieved better Dice compared to the one before applying post-processing. Therefore, in the new Fig. 7 on page 9, we moved the label to the legend.

37. *"The scatter plot illustrates how the optimal parameter set was selected among those with the same performance, prioritising thresholds that preserve more true positives" How is this illustrated in the plot? I would expect that this can be read from the plots. For 12.1 micrometer the smallest variance lower bound seems to be important. But for 2.58 micrometer it is the data point that is in the middle of the plot - Why? Maybe add a sentence like "More true positive values are preserved for parameter sets that [... have the lowest/highest/medium something]."*

Yes, the selection criteria are to preserve as many true positives as possible. We have updated the figure caption to provide more details on how we selected the searched parameter pairs on page 9, Fig.7 caption. Moreover, we also added the reason for choosing search 12 and search 5 in the main text on pages 8 and 10.

- On page 8, line 100-104, we provided the details for choosing search 12 on 2.58 µm data.
- On page 10, line 124-126, we provided the details for choosing search 5 on 12.1 µm data.

Supplement/6 Training on complete organ scans (Fig. 7)

38. *There are errors in the legend with the percentage sign. It shows me an upside down exclamation mark "label volume ¡1% filtered (orange line)", "label volume ¡1% (green line)"*

The figure has been rearranged as Fig.4 on page 6. We have corrected the exclamation mark as "smaller than" in the figure caption on page 6.

39. *Subfigure C: The left plot has no legend and is also styled in a different way than the other plots (grid is more dense), added arrows at x- and y-axis, no box around the plot, no title. I do not really care how you style these, but please make it consistent for one figure (and in the best case for the whole manuscript).*

We have updated Fig. 4 Ci, and the panels within the figure are in the same style.

Supplement/7 Results of LADAF-2021-17 right kidney: "As described in the main manuscript, 5-fold cross-validation of nnUNet on the LADAF-2020-27 left kidney did not show significant variation across folds"

40. *Is this in Fig. 3 in the manuscript? Please be more precise than "in the main manuscript".*

    Yes. The figure that is referred to has been added to this section on page 12, line 149.

Overall/Consistency of figures: I think it is good to have some consistency over all plots that are generated. Examples of inconsistencies I found:

41. *Figure 6 of supplement in (A) it says "v.s." in figure 5 of the supplement it is "vs"*

    The figure has been rearranged as Fig.7 on page 9. We have changed and used 'vs' across the supplementary texts.

42. *Figure 8 in the supplement uses a grey background (all others are white)*

    The figure has been rearranged as Fig.9 on page 12, and the figures have been regenerated and aligned with the style in the main manuscript.

Reviewer #2

This paper proposes a novel multi-scale segmentation pipeline that enables the segmentation of micro-scale functional units across entire organs, thereby facilitating the analysis of functional relationships and spatial distributions within the context of whole-organ anatomy. The proposed framework is innovative, particularly in its use of generated histograms of morphological and spatial properties to filter outliers. The authors conducted extensive experiments and have made both their dataset and code publicly available. This work contributes to the enhanced utilization of HiP-CT and demonstrates significant potential for medical applications. The reviewer encourages the authors to address the following concerns:

Thank you for your insightful suggestions. Please see our responses in the following.

43. *The necessity and effectiveness of fine-tuning and transfer learning are not clear. In terms of feature extraction, the feature space differs substantially across resolutions. The reviewer recommends that the authors provide additional validation regarding the effectiveness of fine-tuning a model trained on high-resolution data when applied to low-resolution data.*

    We provided the experiment results of training from scratch vs fine-tuning on a complete kidney (25.08μm/voxel) in the Supplementary, but we didn't explicitly describe it in the manuscript. To make this comparison clear:

(1) On page 9, lines 251-255, we explicitly list the training from scratch vs fine-tuning in the 'Pseudo-labelling and fine-tuning' section.
(2) In the "Glomeruli segmentation on lower-resolution data" section, we also added a small section at the end to explicitly describe the comparison between fine-tuning and training from scratch on page 13, lines 374-378.

44. *The overfitting behavior observed in UNETR lacks a thorough analysis. As shown in Figure 3, UNETR exhibits more pronounced overfitting compared to the other three methods. The reviewer suggests that the authors include an analysis of the possible causes of this phenomenon.*

We added a new subsection on Page 17, "Model overfitting problem", in the Discussion section. In this subsection, we discussed that the overfitting problem can be caused by a large number of trainable parameters and a lack of long-range dependencies due to the sparse, small annotations.

Reviewer #3

The authors describe a multiscale strategy for deep learning with high, and low resolution images, here HiP-CT scans of the kidney. Their strategy is to mutiscale across scales in the following way: High-resolution predictions are propagated to lower resolutions, which creates training pseudo-labels for models specific to coarser scales. In this way, they are able to achieve segmentation not only at organ-level but also a glomerular levels. This is a promising approach that can move the field forward.

Thank you for your valuable suggestions on our manuscript. We have provided responses as below.

45. *My difficulty in reading the paper was they introduce the various components of their pipeline woven into the numerous sections in the paper. There is no clear outline of the proposed strategy and what networks they use in each stage.*

To improve the reusability of the method section, we tailored the leading paragraph at the beginning of the "Multiscale segmentation pipeline" section on page 6, line 159-172. This paragraph systematically describes Figure 2 of our whole pipeline before diving into each separate steps. In this paragraph, we emphasised that the pipeline employs hierarchical cycles. In the first cycle, we benchmarked four models, and the best-performing one will be selected for subsequent hierarchical cycles.

46. *Further, the terminology "pseudo-labels" was very confusing as the authors never quite define what they mean.*

We have provided more information for ‘pseudo-labels’ in the manuscript. Firstly, in the leading paragraph of the “Multiscale segmentation pipeline” section on page 6, line 159-172, we describe the pseudo-labelling strategy in detail, which uses the predictions of high-resolution VOIs as pseudo-labels in current resolution VOIs.

Secondly, in the ‘Pseudo-labelling and fine-tuning’ section on page 9, line 242-245, at the beginning of this section, we defined the pseudo-labels as the registered predictions from higher-resolution VOIs.

47. *Overall, the paper has a lot of valuable details to offer for the interested practitioner, but it is difficult to read this without first having some bearings as to what the authors wanted to achieve and how did they plan to go about it.*

    We have updated the introduction for clearer clarification. On pages 2 and 3, we restructured paragraphs 3 and 4, line 21 – 51, to better explain what we are going to achieve: proposing a deep learning based multiscale segmentation pipeline for HiP-CT to enable the studies of small functional units across whole organs.
