# Supplementary material for: Multiscale segmentation using hierarchical phase-contrast tomography and deep learning
Source: PLoS Comput Biol. 2026 Feb 2;22(2):e1013923. doi: 10.1371/journal.pcbi.1013923 (PMC12880754; doi:10.1371/journal.pcbi.1013923)
Supplement: Attachment — Submitted filename: review_PLOS.docx [file pcbi.1013923.s002.docx]

Review for “Multiscale Segmentation using Hierarchical Phase-contrast Tomography and Deep Learning”

Overall summary:

The authors present a computational study about kidney segmentation. Especially, they focus on building a tool that can be applied across a multitude of resolutions, because earlier tools can just be applied on the resolution they were trained on. The overall manuscript is well written and seems to be adding something new to the field. I think the manuscript is suitable for publication in PLOS Comp. Bio., but there are concerns from my side that need to be resolved beforehand. These are about:

1. The Dice_I that is introduced in the manuscript
2. The analysis of which models are overfitting
3. The definition/thresholding of the three zones in downstream analysis
4. The availability of the code

# Major:

P. 9 methods/Post-processing/Dice_I

-> I really do not get the point of the Dice_I score. I mean it is just binarizing the Dice score in three ordinal categories. Your argument is that “*precise predictions of the blurry boundaries of glomeruli in low-resolution data are difficult*”. But then the solution should not be to make the evaluation metric “easier” by binarizing, right? I think some discussion about the Dice_I should be included in the discussion or methods section to explain a little bit more why the Dice_I is useful.

-> Also the Dice_I variable is introduced, but never used. It seems like “*Instance Dice scores*” in table 2 is Dice_I. If you introduce the Dice_I you should also use it in your manuscript. E.g. why is it not reported in Table3 anymore?

-> When looking at table 2 the difference between Dice and Dice_I is ~+0.02 for 2.58 and 12.1 before and after correction. For 25.08 there is no notable difference. So is it not enough to just report the Dice? What additional information is given by reporting the Dice_I?

P. 9 methods/Post-processing/Dice_I

“*[...] we propose the DiceI to indicate a higher overlap rate between predictions and ground truths, which helps perform density and distribution studies of the glomeruli on the low-resolution data.*”

-> What do you mean with “*indicate a higher overlap rate*”? I understand it as we correct our Dice score up to have numerically higher results. Wouldn’t it be better to just use the regular Dice score and then explain that worse results are seen because of low-resolution data?

P. 11 results/Glomeruli segmentation on high-resolution labelled data

“*However, for UNETR and SwinUNETR, the training losses decreased while the validation losses increased slightly at the end. Similar patterns were observed in the rise of the Dice scores. These indicated that the UNETR and SwinUNETR experienced the over-fitting problem, achieving better training performance on the training dataset but performing worse on the unseen data.*”

-> I agree that UNETR is overfitting, but SwinUNETR is as much overfitting as nnUNet is. What I mean is that for SwinUNETR AND nnUNet train is larger than validation. I would argue that only UNETR is really overfitting. SwinUNETR is in the same range as nnUNet and still ok in the sense of overfitting.

->You could make this paragraph more precise by actually using numerical differences instead of “*decreases*”, “*increased slightly*”, “*in the rise of*”, “*but performing worse on the*”. This is what I mean:

As training progressed, both the training and validation losses for nnUNet and VNet decreased [BY XXXX%]. However, for UNETR and SwinUNETR, the training losses decreased [BY XXX%] while the validation losses increased slightly [BY XXXX] at the end [AFTER EPOCH XXX]. Similar patterns were observed in the rise [BY XXXXXX] of the Dice scores.

P. 15 results/Morphological analysis

“*Despite the overall uniformity in volume distribution across the cortex, segmenting the cortex into three zones—inner (0–0.33), mid (0.33–0.66), and outer (0.66–1.0)—revealed subtle regional differences.*”

-> These three zones are defined by percentiles and right now I think they lack some more elaborate explanation. Why do you select 3 zones and not e.g. 2 or 4? Either define clearly that they were defined by the 33- and 66-percentile. Alternative and probably more robust way would be to define the number of zones and then the thresholds of the zones by calibration. Also it would be necessary to perform a statistical test to check for the significance of the mean differences. Maybe these zones also need to be defined per patient? For the male kidney I see a peak of glomeruli size between 0.0-0.1 and for the female it is more at ~0.2.

-> The mean glomeruli are in the text around 10**6 micrometer**3. In figure 7, y-axis label it says 10**7, please use the same scale and also add to the y-axis label the unit (what is size of glomeruli, probably micrometer**3).

-> In the discussion (page 18) you name the “*juxtamedullary, mid-cortical, and superficial regions*”. Are those the three zones that you refere to? Maybe try to make this connection more clear. Are there in the literature thresholds for these three zones? Maybe you can use these instead of the 33- and 66-percentile.

-> Add the three zones to the plots (C.1) and (C.2) of figure 7

Github/Code

To summarize: please give some instructions/how-to/getting started to tell me what to do with your code. Right now your codebase is not really accessible which makes the replication of your results very difficult. It also only includes code for preprocessing, no training, no post-processing, and no additional analyses. Here some more specific comments to help you:

-> The documentation of the code needs some work. You provide an empty section about “*Folder structure*” and no instructions about how to set up your repo. I would include a requirements.txt file or conda environment. I just saw that in the *pyproject.toml* there are the dependencies given. Then please provide some instructions on how to set them up.

-> It would also be cool to have a minimal example that is given in the repo to run your code. Maybe just 2 or 3 very small images from your experiments that allow someone to use your functions and make them explorable.

-> Where is some code to make the automatic annotations that you describe in figure 2? I basically only see code relevant for step (B) pre-processing in the repo.

-> Also the post-processing seems to be a relevant step in your work. I do not find any code about the post-processing in the repo. Please add this.

-> please check PLOS policy:<https://journals.plos.org/ploscompbiol/s/code-availability>

# Minor:

P. 6 methods/Data aquisition

-> should be “acquisition”

-> is the “S-20-28” image a private one? It does not have a citation in table1

-> Most kidneys are from France and only one is from Germany. Were they imaged at the same site? So the German kidney was transported to the same facility in France, that’s how I understand this section right now. Please specify.

P. 7 methods/Figure 2

-> “Mannul” please correct this spelling mistake in the figure and also check all other figures for similar spelling errors.

P. 8 methods/data pre-processing

“*Training the deep neural networks on such big datasets is challenging, and 197*

*following preliminary experimentation, we found that conversion of the data from 16-bit 198*

*to 8-bit after application*”

-> Are these preliminary experimentations seen in one of your other papers on this topic (e.g. Vasculature segmentation in 3D hierarchical phase-contrast tomography images of human

Kidneys; Deep learning for 3D vascular segmentation in hierarchical phase contrast tomography: a case study on kidney.)? - If yes please cite.

P. 9 methods/Deep neural network optimisation

-> “*a weighted Dice loss*” what is the weight that is used? In equation 2 and the text I am told that you add the additional Dice loss with a weight beta. You also say that “*beta is the weight of the Dice loss relative to the cross-entropy loss, and is set to 1 by default*” But what is beta then set to during training? Is it the relative occurrence of the classes (glomeruli <-> not-glomeruli)? Is it a fixed value that you adapted during parameter optimization? If already written somewhere else in the manuscript and I could not find it please reference the corresponding section.

P.10 results/Figure 3

-> why do you not have the _train and _val for nnUNet? I think it is really difficult to compare the different models if not the same metric is applied? So either all with moving average on none with moving average. Also this fact should maybe be mentioned somewhere in the methods?

P. 12 results/Table2

Isn’t it weird that the postprocessing is helping so much for the 12.1micrometer case (+~100%), while for the lower and higher resolution the increase is not so much? Can you elaborate this somewhere? Maybe the masking used for 25.08 has an influence? Or it is something else, I am not an expert in kidney segmentation. Maybe the end of this paragraph would be good to add 1 or 2 sentences about this observation? After: “*Dice score and instance Dice scores were highly improved for high and intermediate-resolution data, while slightly improved for the low-resolution complete organ data.*”

P. 16 results/Figure 7

“(A.1) and (A.2) illustrate 3D rendering of predicted glomeruli and the density calculated by a mean kernel of the complete kidney (LADAF-2020-27-Left, Female, aged 94)”

->Define better what is 1 and 2., E.g.:

“*(A.1) illustrates a 3D rendering of predicted glomeruli and (A.2) the density calculated by a mean kernel of the complete kidney (LADAF-2020-27-Left, Female, aged 94)*” -> clearly differentiate between what is 1 and what is 2.

P.17 discussion

“*To address this, we developed an automated post-processing technique at each scale to eliminate false positives [...]*”

-> Did you develop a post-processing pipeline for each scale or did you use different parameters depending on which scale is given. By reading the manuscript (especially Figure 5) I think you have the same pipeline, but with different parameters. But here it sounds like you have different pipelines. Please specify.

P.18 discussion

“*reported that hypertension results in a 20–30% increase in glomerular volume, and our findings are consistent with this trend.*”

-> Could you maybe define your difference between the glomerular volume also in percentage for the three zones between the male and female kidney? This makes it a lot easier to compare your results to the literature (Hughson et al.). Make it easy for the reader and do the math for them, e.g. add “*(zone1: 25%, zone2: 35%, zone3: 45%)*”

Supplement/general

-> The ordering of the sections in the supplement are not in the same order as they occur in the manuscript. Please make this in order. Right now it is 1, 3, 4, 6, 5, 2, 7.

Supplement/1 HiP-CT tomographic information

*“The reconstructed datasets are available for download via the DOI links provided in the Dataset section of the main manuscript”*

-> there are no DOIs in the main manuscript in section Dataset. There are only references given in Table1

Supplement/3 Data pre-processing (Fig. 2)

-> The labels (A) and (B) should be mentioned in the figure description or removed

Supplement/4 Data pre-processing (Fig. 3)

-> The labels (A) and (B) should be mentioned in the figure description or removed

Supplement 5 Prediction post-processing (Fig. 5)

-> Lower panel: red text on orange bars is not readable

-> Also the labels in red and blue text are too long

-> Lower right scatter plot: there seems to be two whitespaces before the “*vs*” in the title

-> X-axis scale of the two scatter plots is not the same, use either full written numbers or 1e6 annotation, but not both.

-> Legend of barplots: write “*Mean Dice_I Score*” to be consistent with the Dice_I that you introduced

-> The scores seem to be categorical (e.g. 12.1 micrometer has only two scores (0.5 and 1.0), is this expected? Looks a little bit artificial.

-> Is the red line really the mean Dice score for 12.1 micrometer? It looks more like the median, because there are only bars on the same level as the mean line and some bars above. Therefore, the mean needs to be larger than 0.5 (maybe ~0.6).

-> “*The scatter plot illustrates how the optimal parameter set was selected among those with the same performance, prioritising thresholds that preserve more true positives*” How is this illustrated in the plot? I would expect that this can be read from the plots. For 12.1 micrometer the smallest variance lower bound seems to be important. But for 2.58 micrometer it is the data point that is in the middle of the plot - Why? Maybe add a sentence like “*More true positive values are preserved for parameter sets that [... have the lowest/highest/medium something].*”

Supplement/6 Training on complete organ scans (Fig. 7)

-> There are errors in the legend with the percentage sign. It shows me an upside down exclamation mark “*label volume ¡1% filtered (orange line)*”, “*label volume ¡1% (green line)*”

-> Subfigure C: The left plot has no legend and is also styled in a different way than the other plots (grid is more dense), added arrows at x- and y-axis, no box around the plot, no title. I do not really care how you style these, but please make it consistent for one figure (and in the best case for the whole manuscript).

Supplement/7 Results of LADAF-2021-17 right kidney

“*As described in the main manuscript, 5-fold cross-validation of nnUNet on the LADAF-2020-27 left kidney did not show significant variation across folds*”

-> Is this in Fig. 3 in the manuscript? Please be more precise than “*in the main manuscript*”.

Overall/Consistency of figures:

I think it is good to have some consistency over all plots that are generated. Examples of inconsistencies I found:

-> Figure 6 of supplement in (A) it says “v.s.” in figure 5 of the supplement it is “*vs*”

-> Figure 8 in the supplement uses a grey background (all others are white)
